# Effectiveness and Cost-Effectiveness of eHealth Interventions in Somatic Diseases: A Systematic Review of Systematic Reviews and Meta-Analyses

**DOI:** 10.2196/jmir.2790

**Published:** 2014-04-16

**Authors:** Niels J Elbert, Harmieke van Os-Medendorp, Wilco van Renselaar, Anne G Ekeland, Leona Hakkaart-van Roijen, Hein Raat, Tamar EC Nijsten, Suzanne GMA Pasmans

**Affiliations:** ^1^Department of (Pediatric) DermatologyErasmus University Medical Center RotterdamRotterdamNetherlands; ^2^Department of Dermatology & AllergologyUniversity Medical Center UtrechtUtrechtNetherlands; ^3^Patiënt1AlmereNetherlands; ^4^Norwegian Centre for Integrated Care & TelemedicineUniversity Hospital of North NorwayTromsøNorway; ^5^Department of Clinical MedicineTelemedicine & e-Health Research GroupUniversity of TromsøTromsøNorway; ^6^Institute for Medical Technology AssessmentErasmus University RotterdamRotterdamNetherlands; ^7^Institute of Health Policy and ManagementErasmus University RotterdamRotterdamNetherlands; ^8^Department of Public HealthErasmus University Medical Center RotterdamRotterdamNetherlands; ^9^Department of Pediatric DermatologyWilhelmina Children’s HospitalUniversity Medical Center UtrechtUtrechtNetherlands

**Keywords:** eHealth, telehealth, telemedicine, review, program effectiveness, cost effectiveness

## Abstract

**Background:**

eHealth potentially enhances quality of care and may reduce health care costs. However, a review of systematic reviews published in 2010 concluded that high-quality evidence on the benefits of eHealth interventions was still lacking.

**Objective:**

We conducted a systematic review of systematic reviews and meta-analyses on the effectiveness/cost-effectiveness of eHealth interventions in patients with somatic diseases to analyze whether, and to what possible extent, the outcome of recent research supports or differs from previous conclusions.

**Methods:**

Literature searches were performed in PubMed, EMBASE, The Cochrane Library, and Scopus for systematic reviews and meta-analyses on eHealth interventions published between August 2009 and December 2012. Articles were screened for relevance based on preset inclusion and exclusion criteria. Citations of residual articles were screened for additional literature. Included papers were critically appraised using the Preferred Reporting Items for Systematic Reviews and Meta-Analyses (PRISMA) Statement before data were extracted. Based on conclusions drawn by the authors of the included articles, reviews and meta-analyses were divided into 1 of 3 groups: suitable, promising, or limited evidence on effectiveness/cost-effectiveness. Cases of uncertainty were resolved by consensus discussion. Effect sizes were extracted from papers that included a meta-analysis. To compare our results with previous findings, a trend analysis was performed.

**Results:**

Our literature searches yielded 31 eligible reviews, of which 20 (65%) reported on costs. Seven papers (23%) concluded that eHealth is effective/cost-effective, 13 (42%) underlined that evidence is promising, and others found limited or inconsistent proof. Methodological quality of the included reviews and meta-analyses was generally considered high. Trend analysis showed a considerable accumulation of literature on eHealth. However, a similar percentage of papers concluded that eHealth is effective/cost-effective or evidence is at least promising (65% vs 62%). Reviews focusing primarily on children or family caregivers still remained scarce. Although a pooled (subgroup) analysis of aggregate data from randomized studies was performed in a higher percentage of more recently published reviews (45% vs 27%), data on economic outcome measures were less frequently reported (65% vs 85%).

**Conclusions:**

The number of reviews and meta-analyses on eHealth interventions in patients with somatic diseases has increased considerably in recent years. Most articles show eHealth is effective/cost-effective or at least suggest evidence is promising, which is consistent with previous findings. Although many researchers advocate larger, well-designed, controlled studies, we believe attention should be given to the development and evaluation of strategies to implement effective/cost-effective eHealth initiatives in daily practice, rather than to further strengthen current evidence.

## Introduction

Willem Einthoven started experiments in 1906 with remote consultations via the telephone network and this is when eHealth is likely to have seen first light [1]. It was not until the 1990s when the number of publications in this field of medicine increased dramatically [2]. This was because of the many studies that were carried out involving remote consultations through video-teleconferencing and digital images to give specialists comparable visual inspection of patients as referring doctors [3].

In modern medical practice, eHealth interventions are increasingly present. With nomenclature evolving rapidly, a significant overlap between terms such as *eHealth*, *telemedicine*, and *telehealth* has occurred. The American Telemedicine Association defines telemedicine as “the use of medical information exchanged from one site to another through electronic communications with the purpose of improving the health status of patients,” and considers *eHealth* and *telehealth* as interchangeable nouns. Both words encompass a broader definition of remote health care and also comprise related services, including nonclinical programs such as education, administration, and research [4]. However, telemedicine is a term that is generally reserved for clinical patient care applications [5].

McLean et al [6] conceptualized the definition of eHealth in a Cochrane review on telehealthcare for asthmatic patients as “the provision of personalized health care at a distance.” eHealth contains the following 3 key elements: (1) data obtained from the patient; (2) electronic transfer of data over a distance; and (3) patient-tailored feedback from a health care professional [5,6]. Therefore, communication in eHealth interventions is personalized and interactive in contrast to patient information websites on health and disease.

eHealth potentially enhances the quality of care and reduces health care costs. It may do so by providing patient education and counseling for primary prevention and early detection of disease, replacing face-to-face visits with health care professionals, collecting patient data on medical parameters remotely, among several other mechanisms [6,7]. Because eHealth interventions are considered complex interventions by the Medical Research Council, difficulty may arise in the assessment of the many interacting components of the intervention [8].

In 2010, Ekeland et al [9] published a systematic review of systematic reviews to evaluate the impact of eHealth interventions on health and health care costs. The authors concluded that high-quality evidence on health and economic benefits was still lacking despite the large number of publications. The primary objective of our review is to analyze whether, and to what possible extent, the outcome of recent research supports or differs from these previous conclusions on the effectiveness/cost-effectiveness of eHealth interventions in patients with somatic diseases.

## Methods

### Overview

Literature searches for systematic reviews and meta-analyses on the effectiveness/cost-effectiveness of eHealth interventions were performed in the following online databases: PubMed, EMBASE, The Cochrane Library, and Scopus. Two of the authors (NE, HO) independently screened all papers’ titles and abstracts for relevance. Citations were screened through Web of Science for additional literature.

### Search Queries

Similar to Ekeland et al [9], we used the following (simplified) search query to retrieve systematic reviews and meta-analyses on the effectiveness of eHealth interventions: “[eHealth] AND [effectiveness] AND [systematic review OR meta-analysis].” To search for papers on cost-effectiveness, “AND [costs]” was added to the aforementioned syntax. Because Ekeland et al [9] took into consideration published works from 2005 to July 2009, we limited our search results to articles published between August 2009 and December 2012. Extensive search queries are presented in Tables 1 and 2.

**Table 1:** PubMed, EMBASE, The Cochrane Library, and Scopus search queries for systematic reviews and meta-analyses on the effectiveness of eHealth interventions (search conducted on September 12, 2013).

Database		Syntax	Hits
**PubMed**			
	Variable 1	e health[Title/Abstract] OR ehealth[Title/Abstract] OR e consultation[Title/Abstract] OR econsultation[Title/Abstract] OR e therapy[Title/Abstract] OR e commerce[Title/Abstract] OR ecommerce[Title/Abstract] OR email consultation[Title/Abstract] OR email consultations[Title/Abstract] OR e mail consultation[Title/Abstract] OR e mail consultations[Title/Abstract] OR telemedicine[Title/Abstract] OR telecare[Title/Abstract] OR teleconsultation[Title/Abstract] OR teleconsultations[Title/Abstract] OR telehealth[Title/Abstract] OR telehomecare[Title/Abstract] OR telehealthcare[Title/Abstract] OR telemonitoring[Title/Abstract] OR telemanagement[Title/Abstract] OR internet[Title/Abstract] OR remote communication[Title/Abstract] OR remote communications[Title/Abstract] OR ict[Title/Abstract] OR web based[Title/Abstract] OR web guided[Title/Abstract]	48,993
	Variable 2	effect[Title/Abstract] OR effects[Title/Abstract] OR effectiveness[Title/Abstract] OR efficiency[Title/Abstract] OR efficacy[Title/Abstract]	4,317,041
	Variable 3	systematic review[Title/Abstract] OR systematic overview[Title/Abstract] OR meta-analysis[Title/Abstract]	76,640
	Total	#1 AND #2 AND #3 AND 2009/01/01[PDat] : 2012/12/31[PDat]	271
**EMBASE**			
	Variable 1	(“e health” OR ehealth OR “e consultation” OR econsultation OR “e therapy” OR “e commerce” OR ecommerce OR “email consultation” OR “email consultations” OR “e mail consultation” OR “e mail consultations” OR telemedicine OR telecare OR teleconsultation OR teleconsultations OR telehealth OR telehomecare OR telehealthcare OR telemonitoring OR telemanagement OR internet OR “remote communication” OR “remote communications” OR ict OR “web based” OR “web guided”):ab,ti	62,242
	Variable 2	(effect OR effects OR effectiveness OR efficiency OR efficacy):ab,ti	5,216,580
	Variable 3	(“systematic review” OR “systematic overview” OR “meta-analysis”):ab,ti	94,419
	Total	#1 AND #2 AND #3 AND (2008-2012)/py	406
**Cochrane Library**			
	Variable 1	(e health OR ehealth OR e consultation OR econsultation OR e therapy OR e commerce OR ecommerce OR email consultation OR email consultations OR e mail consultation OR e mail consultations OR telemedicine OR telecare OR teleconsultation OR teleconsultations OR telehealth OR telehomecare OR telehealthcare OR telemonitoring OR telemanagement OR internet OR remote communication OR remote communications OR ict OR web based OR web guided):ti,ab,kw	15,181
	Variable 2	(effect OR effects OR effectiveness OR efficiency OR efficacy):ti,ab,kw	389,345
	Variable 3	(systematic review OR systematic overview OR meta-analysis):ti,ab,kw	29,734
	Total	(#1 AND #2 AND #3):ti,ab,kw, from 2009 to 2012	385
**Scopus**			
	Variable 1	TITLE-ABS-KEY(“e health” OR ehealth OR “e consultation” OR econsultation OR “e therapy” OR “e commerce” OR ecommerce OR “email consultation” OR “email consultations” OR “e mail consultation” OR “e mail consultations” OR telemedicine OR telecare OR teleconsultation OR teleconsultations OR telehealth OR telehomecare OR telehealthcare OR telemonitoring OR telemanagement OR internet OR “remote communication” OR “remote communications” OR ict OR “web based” OR “web guided”)	365,427
	Variable 2	TITLE-ABS-KEY(effect OR effects OR effectiveness OR efficiency OR efficacy)	11,090,998
	Variable 3	TITLE-ABS-KEY(“systematic review” OR “systematic overview” OR “meta-analysis”)	158,694
	Total	TITLE-ABS-KEY( #1 AND #2 AND #3) AND (LIMIT-TO(PUBYEAR,2012) OR LIMIT-TO(PUBYEAR,2011) OR LIMIT-TO(PUBYEAR,2010) OR LIMIT-TO(PUBYEAR,2009))	595

**Table 2:** PubMed, EMBASE, The Cochrane Library, and Scopus search queries for systematic reviews and meta-analyses on the cost-effectiveness of eHealth interventions (search conducted on September 12, 2013).

Database		Syntax	Hits
**PubMed**			
	Variable 1	e health[Title/Abstract] OR ehealth[Title/Abstract] OR e consultation[Title/Abstract])OR econsultation[Title/Abstract] OR e therapy[Title/Abstract] OR e commerce[Title/Abstract] OR ecommerce[Title/Abstract] OR email consultation[Title/Abstract] OR email consultations[Title/Abstract] OR e mail consultation[Title/Abstract] OR e mail consultations[Title/Abstract] OR telemedicine[Title/Abstract] OR telecare[Title/Abstract] OR teleconsultation[Title/Abstract] OR teleconsultations[Title/Abstract] OR telehealth[Title/Abstract] OR telehomecare[Title/Abstract] OR telehealthcare[Title/Abstract] OR telemonitoring[Title/Abstract] OR telemanagement[Title/Abstract] OR internet[Title/Abstract] OR remote communication[Title/Abstract] OR remote communications[Title/Abstract] OR ict[Title/Abstract] OR web based[Title/Abstract] OR web guided[Title/Abstract]	48,993
	Variable 2	effect[Title/Abstract] OR effects[Title/Abstract] OR effectiveness[Title/Abstract] OR efficiency[Title/Abstract] OR efficacy[Title/Abstract]	4,317,041
	Variable 3	cost[Title/Abstract] OR costs[Title/Abstract] OR economic[Title/Abstract] OR economically[Title/Abstract]	396,949
	Variable 4	systematic review[Title/Abstract] OR systematic overview[Title/Abstract] OR meta-analysis[Title/Abstract]	76,640
	Total	#1 AND #2 AND #3 AND #4 AND 2009/01/01[PDat] : 2012/12/31[PDat]	76
**EMBASE**			
	Variable 1	(“e health” OR ehealth OR “e consultation” OR econsultation OR “e therapy” OR “e commerce” OR ecommerce OR “email consultation” OR “email consultations” OR “e mail consultation” OR “e mail consultations” OR telemedicine OR telecare OR teleconsultation OR teleconsultations OR telehealth OR telehomecare OR telehealthcare OR telemonitoring OR telemanagement OR internet OR “remote communication” OR “remote communications” OR ict OR “web based” OR “web guided”):ab,ti	62,242
	Variable 2	(effect OR effects OR effectiveness OR efficiency OR efficacy):ab,ti	5,216,580
	Variable 3	(cost OR costs OR economic OR economically):ab,ti	502,150
	Variable 4	(“systematic review” OR “systematic overview” OR “meta-analysis”):ab,ti	94,419
	Total	#1 AND #2 AND #3 AND #4 AND (2008-2012)/py	113
**Cochrane Library**			
	Variable 1	(e health OR ehealth OR e consultation OR econsultation OR e therapy OR e commerce OR ecommerce OR email consultation OR email consultations OR e mail consultation OR e mail consultations OR telemedicine OR telecare OR teleconsultation OR teleconsultations OR telehealth OR telehomecare OR telehealthcare OR telemonitoring OR telemanagement OR internet OR remote communication OR remote communications OR ict OR web based OR web guided):ti,ab,kw	15,181
	Variable 2	(effect OR effects OR effectiveness OR efficiency OR efficacy):ti,ab,kw	389,345
	Variable 3	(cost OR costs OR economic OR economically):ti,ab,kw	50,911
	Variable 4	(systematic review OR systematic overview OR meta-analysis):ti,ab,kw	29,734
	Total	(#1 AND #2 AND #3 AND #4):ti,ab,kw, from 2009 to 2012	248
**Scopus**			
	Variable 1	TITLE-ABS-KEY(“e health” OR ehealth OR “e consultation” OR econsultation OR “e therapy” OR “e commerce” OR ecommerce OR “email consultation” OR “email consultations” OR “e mail consultation” OR “e mail consultations” OR telemedicine OR telecare OR teleconsultation OR teleconsultations OR telehealth OR telehomecare OR telehealthcare OR telemonitoring OR telemanagement OR internet OR “remote communication” OR “remote communications” OR ict OR “web based” OR “web guided”)	365,427
	Variable 2	TITLE-ABS-KEY(effect OR effects OR effectiveness OR efficiency OR efficacy)	11,090,998
	Variable 3	TITLE-ABS-KEY(cost OR costs OR economic OR economically)	2,205,295
	Variable 4	TITLE-ABS-KEY(“systematic review” OR “systematic overview” OR “meta-analysis”)	158,694
	Total	TITLE-ABS-KEY( #1 AND #2 AND #3 AND #4) AND (LIMIT-TO(PUBYEAR,2012) OR LIMIT-TO(PUBYEAR,2011) OR LIMIT-TO(PUBYEAR,2010) OR LIMIT-TO(PUBYEAR,2009))	182

### Inclusion Criteria

Systematic reviews and meta-analyses on eHealth interventions in adults and/or children with somatic diseases (ie, illnesses with a physical cause, not mental), and those focusing on family caregivers were included. Interventions had to meet the following 3 criteria: (1) data were obtained from the patient or family caregiver, (2) data were electronically transferred over a distance, and (3) personalized feedback was given from a health care professional. Reviews and meta-analyses of individual studies comparing eHealth interventions to usual or no care, and those comparing different eHealth initiatives were assessed. We only accounted for papers reporting health-related outcomes, costs, patient satisfaction, and/or self-management.

### Exclusion Criteria

Those eHealth interventions that were not home-based (eg, tele-ICU) or not patient or family caregiver–oriented (eg, education of medical or nursing students and health care professionals) were excluded. We excluded meta-analyses that included nonrandomized studies (eg, cohort studies) unless a subgroup analysis of randomized studies (eg, randomized controlled trials, randomized crossover trials) was performed. In addition, we did not assess papers written in languages other than English or Dutch, and those for which the full-text was not available online.

In contrast to Ekeland et al [9], we narrowed the focus of our work by excluding reviews and meta-analyses on nonsomatic disorders (eg, mental disorders such as anxiety, depression, schizophrenia, and posttraumatic stress disorder) and lifestyle changes (eg, smoking cessation and drug intervention programs) to increase the comparability of the included papers and to limit the search results.

### Outcome Measures

Health-related effects (eg, morbidity, mortality, quality of life, hospitalization) and health care costs (eg, health care utilization) were defined as primary outcome measures. We considered patient satisfaction and self-management as secondary outcome measures.

### Critical Appraisal

Before data were extracted, the included papers were critically appraised using the Preferred Reporting Items for Systematic Reviews and Meta-Analyses (PRISMA, formerly QUOROM) Statement [10]. The PRISMA Statement provides an evidence-based 27-item checklist (eg, on objectives, methodology, and limitations) for reporting in systematic reviews and meta-analyses.

### Data Extraction

Based on conclusions drawn by the authors of the included papers, all reviews and meta-analyses were divided into 1 of 3 groups: (1) suitable, (2) promising, or (3) limited evidence that eHealth is effective/cost-effective. Cases of uncertainty were resolved by consensus discussion between 2 authors of the current review (NE, HO). Effect sizes, such as standardized or weighted mean differences, relative risks, odds ratios, and *z* scores, were extracted from papers that included a pooled (subgroup) analysis of aggregate data from randomized studies. No attempt was made to contact authors for missing data. To analyze whether the results of the included papers supported or differed from previous findings by Ekeland et al [9], we performed a trend analysis using basic statistics.

## Results

### Search Results

**Figure 1:**
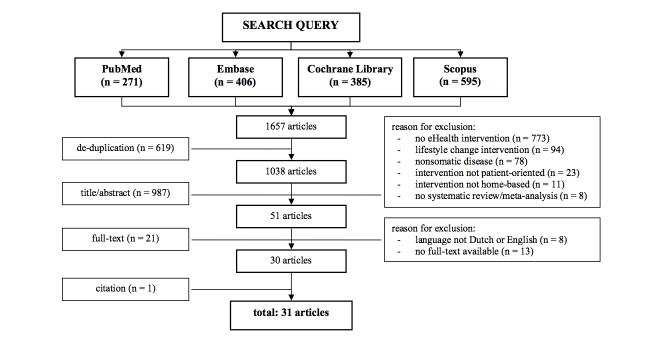
Flow diagram of the literature search on the effectiveness of eHealth interventions.

**Figure 2:**
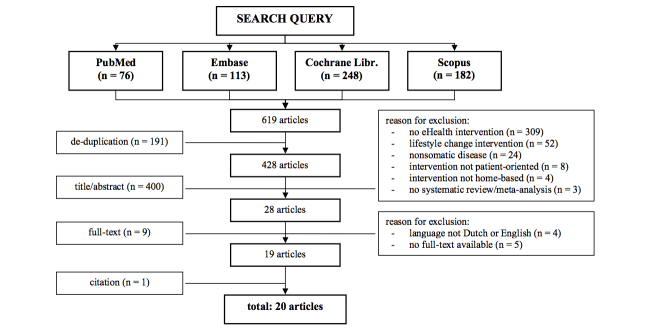
Flow diagram of the literature search on the cost-effectiveness of eHealth interventions.

The initial search yielded a total of 1657 articles, including 619 articles that reported on cost-related outcome measures (Figures 1 and 2). Following removal of duplicates and screening of the residual papers on preset inclusion and exclusion criteria, 30 eligible reviews remained [6,11-39], of which 19 reported on costs [6,13,14,16,18,19,21-24,26,28,29,31,33,34,37-39]. Subsequent citation screening through Web of Science resulted in 1 additional paper [40]. Thus, a total of 31 reviews were retrieved (Figure 1), of which 20 (65%) reported on costs (Figure 2). Three of 31 reviews (10%) reported primarily on children [28,37,38], and 1 of 31 (3%) focused on the effects of eHealth interventions on family caregivers [26].

### Effects of eHealth Interventions

Results per article are summarized in 3 separate tables, 1 for systematic reviews and meta-analyses reporting eHealth interventions are effective/cost-effective (Table 3), a second table for papers showing evidence is promising (Table 4), and a third table with papers underlining evidence is lacking, limited, or inconsistent (Table 5). Table 6 demonstrates the effect sizes-among other characteristics-reported in 14 reviews in which a pooled (subgroup) analysis of aggregate data from randomized studies was performed. All tables are presented subsequently.

### Effectiveness/Cost-Effectiveness of eHealth Interventions

A total of 7 reviews (23%) showed eHealth interventions are effective on either health or cost-related outcome measures (Table 3) [11-17]. Study populations consisted of patients with congestive heart failure (CHF) [13-15,17], diabetes mellitus [12,16], and hypertension [11]. Types of interventions that were effective/cost-effective comprised home telemonitoring [11,13-17], Web or mobile phone-based education [12,16], structured telephone support [14-16], and mobile phone-assisted self-management programs [16]. Patient acceptance and satisfaction were generally considered high.

Pooled analyses were performed in each of the 7 reviews and demonstrated significant reduction of all-cause mortality, all-cause hospitalization, and CHF-related hospital admissions through home telemonitoring and structured telephone support in patients with CHF [13-15,17]. Home telemonitoring also resulted in significant improvement of systolic blood pressure and nonsignificant reduction of diastolic blood pressure, antihypertensive drug use, and therapeutic inertia (ie, unchanged medication despite elevated blood pressure) in hypertensive patients [11]. Web-based education and various mobile phone interventions led to significant improvement of laboratory parameters, such as glycosylated hemoglobin (HbA1c) and low-density lipoprotein (LDL) cholesterol, in diabetic patients [12,16].

Qualitative analysis of individual studies revealed several other positive effects of eHealth interventions, including economic benefits [14,16], reduction of the number of visits to outpatient clinics [12], increase of disease-related knowledge and self-management [12,14,16], and improvement of quality of life [13,14,17].

**Table 3:** Systematic reviews and meta-analyses in which eHealth interventions were shown to be effective/cost-effective.

Study characteristic		Result^a^
**Agarwal et al [11]**		
	Conditions included	Hypertension
	Geographic area	Europe, North America
	Service/intervention	Home telemonitoring
	Outcome measures	Health
	Authors’ summary of results	27 RCTs included comparing home- to office-based blood pressure monitoring. Home TM was used in 7 studies and significantly improved SBP. Meta-analysis also showed reduction of DBP, higher blood pressure response rates, and lower antihypertensive drug use; however, these results were not statistically significant.
	Authors’ conclusions	Home blood pressure monitoring significantly improved SBP compared to office-based measurements. Reductions were even greater when TM was used.
**Angeles et al [12]**		
	Conditions included	Type 1 and 2 diabetes mellitus
	Geographic area	Europe, North America, Asia
	Service/intervention	Web-based education
	Outcome measures	Health, patient satisfaction, self-management
	Authors’ summary of results	9 RCTs included comparing Web-based education to usual care. Meta-analysis showed a significant mean difference in HbA1c after 3, 6, and 12 months and in LDL-cholesterol favoring Web-based education. No significant difference was found for HDL-cholesterol, total cholesterol, and FPG. Other benefits included better patient satisfaction, self-efficacy, and self-management, and reduced clinic visits. However, 1 study demonstrated no differences in health care visits or hospital patient days.
	Authors’ conclusions	Web-based education is superior to usual care in improving HbA1c and LDL-cholesterol
**Clarke et al [13]**		
	Conditions included	Congestive heart failure
	Geographic area	Not stated
	Service/intervention	Home telemonitoring
	Outcome measures	Health, costs, patient satisfaction, self-management
	Authors’ summary of results	13 RCTs included comparing home TM to usual care. Meta-analysis showed significant reduction of all-cause mortality and CHF-related hospital admissions, favoring home TM. No significant difference was found for all-cause hospital and emergency admissions. Qualitative analysis demonstrated no significant difference in hospital length, medication adherence, and costs. 6 studies showed high patient acceptance and satisfaction; 6 different studies reported a trend toward greater improvement of the quality of life. Providing knowledge to the patients allows them to take greater responsibility for their own management and increases patient empowerment.
	Authors’ conclusions	Patients with CHF receiving home TM lived longer without increasing their use of health care facilities. These favorable outcomes support the wider use of home TM.
**Inglis et al [14]**		
	Conditions included	Congestive heart failure
	Geographic area	Europe, North America, Asia, Oceania, Latin America
	Service/intervention	Home telemonitoring, structured telephone support
	Outcome measures	Health, costs, patient satisfaction, self-management
	Authors’ summary of results	25 RCTs included, 11 and 16 of which compared home TM and STS, respectively, to usual care. Meta-analysis showed significant reduction of all-cause mortality, favoring home TM. STS showed a similar, but nonsignificant trend. Both interventions significantly reduced all-cause and CHF-related hospitalization. Qualitative analysis demonstrated reduced costs, high patient satisfaction, and improved quality of life, patient knowledge, and functional class.
	Authors’ conclusions	Home TM and STS appear effective interventions in patients with CHF
**Klersy et al [15]**		
	Conditions included	Congestive heart failure
	Geographic area	Not stated
	Service/intervention	Home telemonitoring, structured telephone support
	Outcome measures	Health
	Authors’ summary of results	32 studies (20 RCTs, 12 cohort studies) were included, comparing home TM and STS to usual care. All-cause mortality and hospital admissions were assessed separately for RCTs and cohort studies; CHF-related hospital admissions could only be assessed for RCTs. Meta-analysis showed significant reduction of all-cause mortality, and all-cause and CHF-related hospitalization, favoring home TM and STS.
	Authors’ conclusions	Home TM and STS confer a significant protective clinical effect, compared to usual care. Mid- and long-term cost-effectiveness of these interventions remains to be evaluated.
**Liang et al [16]**		
	Conditions included	Type 1 and 2 diabetes mellitus
	Geographic area	Not stated
	Service/intervention	Mobile phone interventions, including home telemonitoring, structured telephone support, education and self-management programs
	Outcome measures	Health, costs, patient satisfaction, self-management
	Authors’ summary of results	22 controlled studies (11 RCTs, 2 QRCTs, 2 COTs, 7 NCBAs) were included, assessing the effect of various mobile phone interventions on glycemic control. Meta-analysis showed significant reduction of HbA1c and improvement of self-management, favoring the intervention in both type 1 and 2 diabetic patients. The cost/benefit ratio of the intervention was calculated in only 5 of 22 studies; all 5 reported that the intervention was cost-effective. Most studies reported that the patients were satisfied with the intervention.
	Authors’ conclusions	Mobile phone interventions effectively improve glycemic control and self-management in diabetic patients, especially in patients with type 2 diabetes mellitus
**Polisena et al [17]**		
	Conditions included	Congestive heart failure
	Geographic area	Europe, North America, Asia
	Service/intervention	Home telemonitoring
	Outcome measures	Health, patient satisfaction
	Authors’ summary of results	21 studies (11 RCTs, 6 cohort studies, 4 NUBAs) included comparing home TM to usual care. Meta-analysis of the included RCTs showed significant reduction of all-cause mortality, favoring home TM. Qualitative analysis suggests home TM may lower hospitalization rates and the use of other health care services. Quality of life and patient satisfaction with home TM were similar or better than with usual care.
	Authors’ conclusions	Home TM is clinically effective, but the effect on health care utilization is more limited

^a^CHF: congestive heart failure; COT: randomized crossover trial; DBP: diastolic blood pressure; FPG: fasting plasma glucose; HbA1c: glycosylated hemoglobin; HDL: high-density lipoprotein; LDL: low-density lipoprotein; NCBA: nonrandomized controlled before-after study; NUBA: nonrandomized uncontrolled before-after study; QRCT: quasi-randomized controlled trial; RCT: randomized controlled trial; SBP: systolic blood pressure; STS: structured telephone support; TM: telemonitoring.

### Evidence on eHealth Interventions is Promising

Thirteen reviews (42%) were less confident about the effectiveness/cost-effectiveness of eHealth interventions [18-29,40], but suggested that these initiatives are promising or bear potential (Table 4). Many of the authors claim additional research is needed to clarify efficacy and cost-related issues.

Pooled analyses were performed in 4 reviews and presented subsequently [22-24,27]. One review on chronic obstructive pulmonary disease (COPD) demonstrated the capacity of eHealth interventions to significantly reduce the number of patients with 1 or more emergency department visits or hospital admissions-due to exacerbation of pulmonary symptoms-over a 12-month period [23]. eHealth interventions did not significantly improve quality of life and all-cause mortality. Because the interventions were often part of complex interventions, the authors concluded that further investigation is required to determine the precise role of eHealth. Promising effects were also identified for Internet-based peer and clinical visit support programs—among several other eHealth interventions—in acute and chronic pain management [18,22]. Although the Internet was supportive in the treatment of pain, it remained unclear what benefits could be gained and which patients would profit most.

**Table 4:** Systematic reviews and meta-analyses in which promising evidence on the effectiveness/cost-effectiveness of eHealth interventions was reported.

Study characteristic		Result^a^
**Bender et al [18]**		
	Conditions included	Acute and chronic pain
	Geographic area	North America
	Service/intervention	Internet-based peer and clinical visit support programs, including education and self-management programs
	Outcome measures	Health, costs, patient satisfaction, self-management
	Authors’ summary of results	17 RCTs were included, of which 6 compared Internet-based peer and clinical visit support programs to usual care or an existing nursing website; the other 11 articles described cognitive and behavioral interventions. Meta-analysis was considered inappropriate due to substantial heterogeneity among studies. Qualitative analysis showed limited but promising evidence that Internet-based peer support programs can lead to improvements of pain intensity, activity limitations, health distress and self-management, and can reduce pain in children and adolescents. Insufficient evidence was found on the effects of Internet-based clinical support interventions. Two studies found no significant difference in health care utilization; another study showed significant difference in knowledge and patient satisfaction.
	Authors’ conclusions	Internet-based interventions seem promising for people in pain, but it remains unclear which patients benefit most
**Eland-de Kok et al [19]**		
	Conditions included	Chronic diseases
	Geographic area	Not stated
	Service/intervention	Interactive websites with store-and-forward services, including home telemonitoring, video-teleconferencing, education, self-management programs and cardiac rehabilitation
	Outcome measures	Health, costs, patient satisfaction
	Authors’ summary of results	12 RCTs included assessing interactive websites with store-and-forward services as an addition to or instead of usual face-to-face care. Meta-analysis was considered inappropriate due to substantial heterogeneity among studies. Qualitative analysis showed significant improvement of physical health outcomes with small to moderate effect sizes. Not all outcomes improved, and some measures showed comparable effect sizes. Costs, quality of life, and patient satisfaction were rarely assessed and showed various results.
	Authors’ conclusions	eHealth is a promising tool for treatment and self-management training of chronically ill patients
**Hailey et al [20]**		
	Conditions included	Any
	Geographic area	Not stated
	Service/intervention	In-home telerehabilitation
	Outcome measures	Health, self-management
	Authors’ summary of results	61 studies with various types of study designs (not further specified) were included. For a variety of populations and types of outcome, 71% of the interventions were successful, 18% were unsuccessful, and for 11% the status was unclear. The reported outcomes for 51% of the interventions appeared to be clinically significant. Success was not demonstrated in cardiac studies on improvements in self-efficacy and physical activity.
	Authors’ conclusions	In-home telerehabilitation shows promise in many fields, but compelling evidence of benefit is still limited
**Johansson et al [21]**		
	Conditions included	Stroke
	Geographic area	Europe, North America, Asia
	Service/ intervention	In-home telerehabilitation, including telephone and videophone consulting
	Outcome measures	Health, costs, patient satisfaction
	Authors’ summary of results	9 studies (4 RCTs, 4 case series, 1 qualitative analysis) were included. Qualitative analysis showed better SF-36 scores in stroke patients who underwent in-home telerehabilitation, but this difference was not significant. No significant differences were found in various secondary outcome measures such as the Hospital Anxiety and Depression Scale, Barthel Index, modified Ranking Scale, and the use of secondary prevention drugs since discharge. Participants reported high level of satisfaction and acceptance of the intervention. No study reported on cost-effectiveness or resource utilization.
	Authors’ conclusions	In-home telerehabilitation showed promising results in poststroke care, but the quality of evidence was low
**McGeary et al [22]**		
	Conditions included	Chronic pain
	Geographic area	Not stated
	Service/intervention	Technology-based interventions, including structured telephone support, video-teleconferencing, self-management programs, and outpatient telerehabilitation
	Outcome measures	Health, costs, patient satisfaction, self-management
	Authors’ summary of results	10 studies (9 RCTs, 1 COT) were included, comparing a range of technologies to usual care or waiting-list control conditions. Meta-analysis showed a significant overall benefit of eHealth interventions over control conditions and equivalence with in-person interventions.
	Authors’ conclusions	eHealth interventions can result in successful pain management, but qualitative trials are lacking. Therefore, it is unclear exactly what benefits can be obtained.
**McLean et al [23]**		
	Conditions included	COPD
	Geographic area	Europe, North America, Asia
	Service/intervention	Telehealthcare interventions, including home telemonitoring, structured telephone support, video-teleconferencing and self-management programs
	Outcome measures	Health, costs, patient satisfaction, self-management
	Authors’ summary of results	10 RCTs were included, comparing a range of telehealthcare interventions to usual care or face-to-face home visits. Meta-analysis showed significant reduction of all-cause emergency department visits and hospital admissions, favoring eHealth. Quality of life and all-cause mortality did not significantly improve. Three studies reported on patient satisfaction using different invalidated scales.
	Authors’ conclusions	Telehealthcare interventions appear to have a possible effect on quality of life and all-cause emergency department visits and hospital admissions. Further research is needed to clarify precisely its role, since interventions were assessed as part of a complex intervention.
**Omboni et al [24]**		
	Conditions included	Hypertension
	Geographic area	Not stated
	Service/intervention	Home telemonitoring, education
	Outcome measures	Health, costs
	Authors’ summary of results	12 RCTs were included, comparing home blood pressure TM to usual care. 6 interventions incorporated an educational component. Meta-analysis showed significant improvement of blood pressure control and reduction of both SBP and DBP, favoring home TM. Home TM was associated with a modest, but significantly increased use of antihypertensive medications. Information on costs was available from merely a few studies. In 1 study, quality of life tended to be higher and costs lower in the intervention group teletransmitting blood pressure data. Another study observed lower medication and consultation costs in the intervention group, which were however offset by the cost of the telemonitoring equipment.
	Authors’ conclusions	Home TM may represent a useful tool to improve blood pressure control and reduce adverse cardiovascular events. Well-designed, large-scale RCTs are still needed to demonstrate its superiority and clinical usefulness.
**Paré et al [25]**		
	Conditions included	Chronic diseases
	Geographic area	Europe, North America, Asia
	Service/intervention	Home telemonitoring, education
	Outcome measures	Health
	Authors’ summary of results	62 studies (45 RCTs, 17 nonrandomized studies that were not further specified) were included to assess the clinical effects of home TM. Meta-analysis was considered inappropriate due to substantial heterogeneity among studies. Qualitative analysis of studies on diabetes mellitus indicated a trend toward better glycemic control with home TM. In most trials on asthma, significant improvement of peak expiratory flows and quality of life, and reduction of asthma-related symptoms were found. Studies on hypertension generally demonstrated reduction of SBP and/or DBP.
	Authors’ conclusions	Home TM appears to be promising in patient management, but designers of future studies should consider ways to make this technology more effective as well as controlling possible mediating variables
**Pron et al [40]**		
	Conditions included	Cardiac arrhythmia, heart failure
	Geographic area	Europe, North America
	Service/intervention	Internet-based device-assisted remote monitoring systems for therapeutic cardiovascular implantable electronic devices
	Outcome measures	Health, costs, patient satisfaction
	Authors’ summary of results	23 studies (7 RCTs, 16 cohort studies) were included, comparing Internet-based device-assisted RMSs to usual care. Qualitative analysis of multiple cohort studies and 2 RCTs demonstrated feasibility and significant reduction of in-office clinic follow-ups with RMSs in the first year post implantation. Detection rates of clinically significant events were higher and the time to a clinical decision for these events was significantly shorter. Earlier detection was not associated with lower morbidity or mortality rates. Patient acceptance and satisfaction were reported to be high. The incremental cost of providing RMSs was approximately Can –$409K per year (cost savings); corresponding incremental cost per patient was Can –$98 per year.
	Authors’ conclusions	RMSs have the potential to improve current surveillance systems, but there is insufficient information to evaluate the overall impact to the health care system
**Rietdijk et al [26]**		
	Conditions included	Traumatic brain injury
	Geographic area	Not stated
	Service/intervention	Technology-assisted training and support programs, including video-teleconferencing, education and self-management interventions
	Outcome measures	Health, costs, patient satisfaction
	Authors’ summary of results	16 studies (7 RCTs, 4 CCTs, 5 case series) were included, qualitatively describing the effectiveness of technology-assisted training and support programs to family caregivers of patients with traumatic brain injury. Meta-analysis was considered inappropriate due to substantial heterogeneity among studies. All but 1 study reported some degree of positive results on feasibility, user satisfaction, and preliminary explorations of effectiveness. No studies reported any formal cost analysis, although 1 study that provided cost estimates noted the intervention was much less expensive than the intensive inpatient comparison condition which had similar outcomes.
	Authors’ conclusions	Technology-assisted programs seem a promising approach in the training and support of family members of patients with traumatic brain injury
**Samoocha et al [27]**		
	Conditions included	Any
	Geographic area	Not stated
	Service/intervention	Web-based interventions, including education and self-management programs
	Outcome measures	Self-management
	Authors’ summary of results	14 RCTs included comparing Web-based education to usual care, waiting-list control conditions, or no care in various somatic conditions. Meta-analysis showed a significant positive effect on patient empowerment measured with the Diabetes Empowerment Scale and on self-efficacy measured with disease-specific scales, both favoring Web-based tools. No effects were found for self-efficacy measured with general scales.
	Authors’ conclusions	Web-based education showed positive, but generally small effects. Direct and indirect impacts of these effects remain unknown
**Stinson et al [28]**		
	Conditions included	Chronic diseases
	Geographic area	Europe, North America, Asia
	Service/intervention	Internet-based self-management programs
	Outcome measures	Health, costs, self-management
	Authors’ summary of results	9 studies (7 RCTs, 1 pilot RCT, 1 quasi-experimental study) with Internet-based self-management programs were included. Due to the limited data reported in the studies only a qualitative analysis was provided. Seven studies demonstrated significant improvement of health-related outcome measures, compared to the control group. There was conflicting evidence regarding the effect of Internet-based self-management programs on disease-specific knowledge and quality of life, whereas evidence on health care utilization was limited.
	Authors’ conclusions	The Internet shows great promise as a mode of delivering self-management programs for youth with health conditions
**Van den Berg et al [29]**		
	Conditions included	Elderly people with chronic diseases
	Geographic area	Europe, North America, Asia, Oceania, Latin America
	Service/intervention	Telemedicine interventions, including home telemonitoring, structured telephone support, video-teleconferencing, education, self-care training and telerehabilitation
	Outcome measures	Health, costs, patient satisfaction, self-management
	Authors’ summary of results	68 studies (56 RCTs, 12 CCTs) were included, a range of technologies to one another or to usual care in elderly people with chronic diseases. Literature shows predominantly positive results with a clear trend toward better results for telemedicine interventions, independent of the diagnosis group. 36 studies comprised an economic endpoint, of which 15 showed positive and 2 mixed results. None of the studies reported a significantly better outcome for the control group.
	Authors’ conclusions	The many positive examples provided in the literature indicate the considerable potential of eHealth

^a^CCT: nonrandomized controlled clinical trial; COPD: chronic obstructive pulmonary disease; COT: randomized crossover trial; DBP: diastolic blood pressure; RCT: randomized controlled trial; RMS: remote monitoring systems; SBP: systolic blood pressure; SF: short form; TM: telemonitoring.

Qualitative analysis of individual studies revealed many other promising effects of eHealth interventions, for example, Internet-based device-assisted remote monitoring systems in patients with cardiovascular implantable electronic devices [40], in-home telerehabilitation in routine care of patients with stroke and other somatic diseases [20,21], technology-assisted training and support programs for family members of patients with traumatic brain injury [26], and Web-based education to increase patient empowerment [27]. Paré et al [25] assessed the clinical effects of home telemonitoring in patients with a variety of chronic diseases. The authors highlight the fact that home telemonitoring allows for closer follow-up of individual patients’ conditions and for early detection of warning signs in case of health deterioration. However, they claim larger trials are needed to confirm the clinical effects of home telemonitoring.

### Evidence on eHealth Interventions Is Lacking, Limited or Inconsistent

Eleven reviews (35%) underlined that evidence on the effectiveness/cost-effectiveness of eHealth interventions is still lacking, limited, or inconsistent (Table 5) [6,30-39]. In many articles, the poor methodological quality of individual studies is criticized, and ambiguous or conflicting findings are emphasized.

McLean et al [6] conducted a Cochrane review of 21 RCTs on a range of eHealth interventions in patients with asthma. Meta-analysis did not show a clinically important improvement of disease-specific quality of life, and no significant reduction of all-cause emergency department visits over a 12-month period was found (Table 6). The authors concluded that eHealth is unlikely to result in clinically relevant improvements of health-related outcome measures in patients with relatively mild disease, but does appear to have the potential to reduce all-cause hospital admissions in those with more severe disease.

Shulman et al [37] studied the impact of eHealth interventions involving transmission of blood glucose data in youth with type 1 diabetes mellitus. Pooled analyses showed no apparent effect of the interventions on HbA1c or acute complications, such as severe hypoglycemia and diabetic ketoacidosis (Table 6). The limited data available on patient satisfaction and costs also suggested no differences between the intervention and the comparison group.

**Table 5:** Systematic reviews and meta-analyses in which no, limited, or inconsistent evidence on the effectiveness/cost-effectiveness of eHealth interventions was reported.

Study characteristic		Result^a^
**Baron et al [30]**		
	Conditions included	Type 1 and 2 diabetes mellitus
	Geographic area	Europe, North America, Asia
	Service/ intervention	Mobile phone telemonitoring
	Outcome measures	Health
	Authors’ summary of results	20 studies (12 RCTs, 4 NUBAs, 2 pilot COTs, 1 NCBA, 1 pilot NUBA) were included. Of the 15 controlled studies, 9 compared mobile phone telemonitoring to standard care and 6 to a different (eHealth) intervention. Qualitative analysis demonstrated mixed results of diet-focused interventions. Evidence on the effect of nondietary interventions in patients with type 1 diabetes mellitus was inconclusive. Of the 13 studies reporting on patients with type 2 diabetes mellitus, 7 found structured mobile phone support to be more effective than other eHealth interventions and standard care in reducing HbA1c.
	Authors’ conclusions	Evidence on the effectiveness of mobile phone telemonitoring was inconsistent and remains weak
**Bolton et al [31]**		
	Conditions included	COPD
	Geographic area	Not stated
	Service/ intervention	Home telemonitoring, education
	Outcome measures	Health, costs
	Authors’ summary of results	6 studies (2 RCTs, 2 NCBAs, 2 NUBAs) on home TM were included, of which some had an educational element. Meta-analysis was considered inappropriate, because individual studies were underpowered, had heterogeneous patient populations and had a lack of detailed intervention description. Qualitative analysis showed positive results on health and costs.
	Authors’ conclusions	The benefit of home TM in patients with COPD is not yet proven and further research is required before large-scale implementation
**Ciere et al [32]**		
	Conditions included	Congestive heart failure
	Geographic area	Europe, North America
	Service/ intervention	Telehealthcare interventions, including home telemonitoring and education
	Outcome measures	Self-management
	Authors’ summary of results	12 studies (9 RCTs, 1 pilot RCT, 1 CCT, 1 NUBA) were included, comparing various telehealthcare interventions to usual care or to home nurse visits. Meta-analysis was considered inappropriate due to substantial heterogeneity among studies. Qualitative analysis showed inconclusive evidence that telehealthcare interventions improve patient knowledge, self-care, or self-efficacy.
	Authors’ conclusions	Literature provides insufficient evidence to robustly support or disprove beneficial effects of telehealthcare interventions in patients with CHF
**Franek [33]**		
	Conditions included	COPD
	Geographic area	Not stated
	Service/ intervention	Home telemonitoring, structured telephone support
	Outcome measures	Health, costs, patient satisfaction, self-management
	Authors’ summary of results	5 studies (3 RCTs, 2 CCTs) were included, comparing home TM to usual care in patients with moderate to severe COPD; another RCT compared STS to usual care. Meta-analysis was considered inappropriate due to substantial heterogeneity among studies. Qualitative analysis showed nonsignificant or conflicting effects of home TM for all outcome measures, including health care utilization, mortality, quality of life, number of exacerbations, patient satisfaction, and safety. Low quality evidence showed significant benefit in favor of STS for self-efficacy and emergency department visits, but nonsignificant results for hospitalization and hospital length of stay. The economic impact of both interventions was uncertain.
	Authors’ conclusions	Low to very low quality evidence found nonsignificant or conflicting effects of home TM for all outcome measures. Low quality evidence showed significant benefit of STS for self-efficacy and emergency department visits, but nonsignificant results for hospitalization and hospital length of stay.
**McLean et al [6]**		
	Conditions included	Asthma
	Geographic area	Europe, North America, Asia, Oceania, Latin America
	Service/ intervention	Telehealthcare interventions, including home telemonitoring, structured telephone support, video-teleconferencing, education and self-management programs
	Outcome measures	Health, costs, patient satisfaction
	Authors’ summary of results	21 RCTs were included, comparing a range of technologies to (enhanced) face-to-face usual care. Meta-analysis showed no clinically important improvement of disease-specific quality of life, and no significant reduction of all-cause emergency department visits over 12 months was found. However, all-cause hospital admissions over 12 months were significantly reduced, particularly in patients with severe asthma. Costs were favorable to continuing the intervention where hospitalization was prevented, but this was not true for all studies.
	Authors’ conclusions	Telehealthcare interventions are unlikely to result in clinically relevant improvements in patients with relatively mild asthma, but do appear to have the potential to reduce all-cause hospital admissions in those with more severe disease
**Mistry [34]**		
	Conditions included	Any
	Geographic area	Predominantly Europe and North America
	Service/ intervention	Telemedicine and telecare interventions not otherwise specified
	Outcome measures	Costs
	Authors’ summary of results	80 studies (38 CCAs, 18 CMAs, 15 CEAs, 7 CUAs, 2 CBAs) were included. Economic tools are increasingly being used to evaluate telemedicine and telecare interventions, but transparency in the reporting of methodologies and results is required. Literature showed no general agreement whether eHealth interventions were cost-effective, compared to conventional means.
	Authors’ conclusions	Literature provides no conclusive evidence on the cost-effectiveness of telemedicine and telecare interventions
**Ryhänen et al [35]**		
	Conditions included	Breast cancer
	Geographic area	North America
	Service/ intervention	Internet or interactive computer-based education
	Outcome measures	Health, patient satisfaction, self-management
	Authors’ summary of results	14 studies (9 RCTs, 2 CCTs, 3 quasi-experimental studies) were included, comparing Internet or interactive computer-based education to various control conditions, such as usual or no care, waiting-list control conditions, and discussion with counselors or physicists. Meta-analysis was considered inappropriate due to substantial heterogeneity among studies. Literature suggests that Internet or interactive computer-based education increase patients’ knowledge and health information competence, and positively affect patient satisfaction.
	Authors’ conclusions	No clear effect on patient outcome measures could be identified, because effects differed across studies
**Saksena [36]**		
	Conditions included	Hypertension
	Geographic area	Europe, North America, Asia
	Service/ intervention	Computer-based education
	Outcome measures	Health, self-management
	Authors’ summary of results	5 studies (4 RCTs, 1 CCT) were included, assessing the effects of computer-based education on knowledge, self-management, and blood pressure control. Different control conditions were used, including usual care, pamphlet and website registration, and searching Yahoo. Meta-analysis was considered inappropriate due to substantial heterogeneity among studies. Quantitative analysis showed significant improvement of knowledge in 3 studies; self-management improved significantly in another study. Only 1 study demonstrated a significant increase of patients with controlled blood pressure before and after the intervention.
	Authors’ conclusions	Computer-based education is insufficient to replace provider-based education
**Shulman et al [37]**		
	Conditions included	Type 1 diabetes mellitus
	Geographic area	Not stated
	Service/ intervention	Home telemonitoring, education
	Outcome measures	Health, costs, patient satisfaction
	Authors’ summary of results	10 RCTs were included, comparing a range of technologies involving transmission of blood glucose data followed by unsolicited scheduled clinician feedback to usual care in youth with type 1 diabetes mellitus. Some studies incorporated an educational co-intervention. Meta-analysis showed no significant effect of the interventions on HbA1c, severe hypoglycemia or diabetic ketoacidosis. Limited data on patient satisfaction, quality of life, and costs also suggest no group differences.
	Authors’ conclusions	It is unlikely that eHealth interventions have a substantial effect on glycemic control or acute complications
**Welsh et al [38]**		
	Conditions included	Asthma
	Geographic area	North America, Oceania
	Service/ intervention	Home-based education
	Outcome measures	Health, costs
	Authors’ summary of results	12 RCTs were included, comparing home-based education to usual care in children with asthma. Meta-analysis showed no significant difference in the mean number of exacerbations requiring emergency department visits. Narrative analysis demonstrated improvement of quality of life in both groups over time. None of the studies analyzed cost-effectiveness.
	Authors’ conclusions	Inconsistent evidence was found for home-based education compared to usual care
**Wootton [39]**		
	Conditions included	Chronic diseases
	Geographic area	Not stated
	Service/intervention	Telemedicine interventions, including home telemonitoring, structured telephone phone support, video-teleconferencing, education and self-management programs
	Outcome measures	Health, costs, self-management
	Authors’ summary of results	In total, 141 RCTs were included. Meta-analysis was considered inappropriate due to substantial heterogeneity among studies. Most studies reported positive or weakly positive effects, and almost none reported negative effects. There was no significant difference in effect between diagnosis groups. There have been very few studies on cost-effectiveness.
	Authors’ conclusions	The evidence base for the value of eHealth is generally weak and contradictory

^a^CBA: cost-benefit analysis.; CCA: cost-consequences analysis; CCT: nonrandomized controlled clinical trial; CEA: cost-effective analysis; CHF: congestive heart failure; CMA: cost-minimization analysis; COPD: chronic obstructive pulmonary disease; COT: randomized crossover trial; CUA: cost-utility analysis; HbA1c: glycosylated hemoglobin; NCBA: nonrandomized controlled before-after study; NUBA: nonrandomized uncontrolled before-after study; RCT: randomized controlled trial; STS: structured telephone support; TM: telemonitoring.

**Table 6:** Characteristics of 14 systematic reviews in which a meta-analysis was performed.

Study		Design (n)^a^	Condition^b^	Intervention^c^	Control^c^	Outcome^d^	Effect size (95% CI)^e^	Heterogeneity^f^
**Meta-analyses reporting eHealth interventions are effective/cost-effective**								
	Agarwal et al [11]	RCT (5)	HT	Home TM	Office-based monitoring	SBP	SMD=–3.20 (–4.66, –1.73)	NR
						DBP	SMD=–1.63 (–2.47, –0.79)	NR
						Blood pressure response	RR=1.17 (1.02, 1.34)	NR
						Antihypertensive drug use	RR=2.18 (0.20, 23.68)	NR
	Angeles et al [12]	RCT (9)	Type 1 and 2 DM	Web-based education	Usual care	HbA1c	3 m: WMD=–0.71 (–1.00, –0.43)/6 m: WMD=–0.52 (–0.75, –0.29)/12 m: WMD=–0.55 (–0.70, –0.39)	0/0/78
						FPG	3 m: WMD=–0.47 (–1.30, 0.35)/12 m: WMD=–0.80 (–2.81, 1.20)	0/85
						LDL-cholesterol	WMD=–0.23 (–0.28, –0.19)	11
						HDL-cholesterol	WMD=–0.00 (–0.15, 0.15)	83
						Total cholesterol	WMD=–0.14 (–0.53, 0.25)	70
	Clarke et al [13]	RCT (13)	CHF	Home TM	Usual care	All-cause mortality	RR=0.77 (0.61, 0.97)	51
						All-cause hospital admissions	RR=0.99 (0.88, 1.11)	59
						CHF-related hospital admissions	RR=0.73 (0.62, 0.87)	0
						All-cause ED visits	RR=1.04 (0.86, 1.26)	82
	Inglis et al [14]	RCT (11)	CHF	Home TM	Usual care	All-cause mortality	RR=0.66 (0.54, 0.81)	0
						All-cause hospital admissions	RR=0.91 (0.84, 0.99)	78
						CHF-related hospital admissions	RR=0.79 (0.67, 0.94)	39
		RCT (16)	CHF	STS	Usual care	All-cause mortality	RR=0.88 (0.76, 1.01)	0
						All-cause hospital admissions	RR=0.92 (0.85, 0.99)	24
						CHF-related hospital admissions	RR=0.77 (0.68, 0.87)	7
	Klersy et al [15]	RCT (20)	CHF	Home TM, STS	Usual care	All-cause mortality	RR=0.83 (0.73, 0.95)	0
						All-cause hospital admissions	RR=0.93 (0.87, 0.99)	18
						CHF-related hospital admissions	RR=0.71 (0.64, 0.80)	2
	Liang et al [16]	RCT (11)	Type 1 and 2 DM	Mobile phone interventions	NR	HbA1c	SMD=0.5 (0.2, 0.8)	NR
	Polisena et al [17]	RCT (11)	CHF	Home TM	Usual care	All-cause mortality	RR=0.60 (0.45, 0.81)	0
**Meta-analyses reporting promising evidence on effectiveness/cost-effectiveness of eHealth interventions**								
	McGeary et al [22]	RCT (9), COT (1)	Chronic pain	STS, VTC, self-management programs and outpatient telerehabilitation	Usual care or waiting list	Pain intensity ratings	*z*=–4.74 (–0.9, –0.4)	Q=15.73 (*P*=.07)
	McLean et al [23]	RCT (10)	COPD	Home TM, STS, VTC and self-management programs	Usual care or face-to-face home visits	All-cause mortality	OR=1.05 (0.63, 1.75)	0
						All-cause hospital admissions	OR=0.46 (0.33, 0.65)	0
						All-cause ED visits	OR=0.27 (0.11, 0.66)	77
						Quality of life^g^	SMD=–6.57 (–13.62, 0.48)	51
	Omboni et al [24]	RCT (12)	HT	Home TM, education	Usual care	SBP	WMD=5.64 (7.92, 3.36)	66
						DBP	WMD=2.78 (3.93, 1.62)	57
						Blood pressure control	RR=1.31 (1.06, 1.62)	78
						Antihypertensive drug use	WMD=0.22 (0.02, 0.43)	79
	Samoocha et al [27]	RCT (14)	Any	Web-based education and self-management programs	Usual care, waiting list, or no care	Empowerment^h^	SMD=0.61 (0.29, 0.94)	0
						Self-efficacy (disease-specific)	SMD=0.23 (0.12, 0.33)	27
						Self-efficacy (general)	SMD=0.05 (–0.25, 0.35)	27
**Meta-analyses reporting lacking, limited or inconsistent evidence on the effectiveness/cost-effectiveness of eHealth interventions**								
	McLean et al [6]	RCT (21)	Asthma	Home TM, STS, VTC, education and self-management programs	(Enhanced) face-to-face usual care	All-cause hospital admissions	3 m: OR=0.47 (0.01, 36.46)/12 m: OR=0.21 (0.07, 0.61)	84/0
						All-cause ED visits	OR=1.16 (0.52, 2.58)	29
						Quality of life^i^	WMD=0.08 (0.01, 0.16)	24
	Shulman et al [37]	RCT (10)	Type 1 DM	Home TM, education	NR	HbA1c	WMD=–0.12 (–0.35, 0.11)	0
						Severe hypoglycemia	OR=1.42 (0.22, 9.32)	0
						Diabetic ketoacidosis	OR=1.02 (0.24, 4.23)	0
	Welsh et al [38]	RCT (12)	Asthma	Home-based education	Usual care	Asthma-related ED visits	6 m: WMD=0.04 (–0.20, 0.27)/12-18 m: WMD=–0.32 (–0.74, 0.10)	NR/NR

^a^RCT: randomized controlled trial; COT: randomized crossover trial.

^b^HT: hypertension; CHF: congestive heart failure; DM: diabetes mellitus; COPD: chronic obstructive pulmonary disease.

^c^TM: telemonitoring; STS: structured telephone support; VTC: video-teleconferencing; NR: not reported.

^d^SBP: systolic blood pressure; DBP: diastolic blood pressure; HbA1c: glycosylated hemoglobin; FPG: fasting plasma glucose; LDL: low-density lipoprotein; HDL: high-density lipoprotein; ED: emergency department.

^e^SMD: standardized mean difference also known as Cohen’s *d*; a conventional rule is to consider a Cohen’s *d* of 0.2 as small, 0.5 as medium, and 0.8 as large [41]; RR: relative risk; WMD: weighted mean difference.

^f^Unless otherwise indicated, data are presented as a percentage of variation across studies that is due to heterogeneity (ie, *I* ^*2*^ statistic). An *I* ^*2*^ value greater than 50 is considered as substantial heterogeneity and may indicate that quantitative analysis is inappropriate [17,42].

^g^Using the St. George’s Respiratory Questionnaire.

^h^Using the Diabetes Empowerment Scale.

^i^Using the Juniper Asthma Quality of Life Questionnaire.

### Methodological Quality of Reviews and Meta-Analyses

Among the systematic reviews and meta-analyses described in the current review are 4 high-quality Cochrane reviews [6,14,23,38]. Following the PRISMA Statement [10], the methodological quality of the other included papers was generally considered high. Nearly all authors provided search queries and selection criteria, described the process of data extraction, presented the results and limitations of individual studies, and demonstrated the implications of their outcome for daily practice and future research. If the authors received external funding, this was reported.

Some discrepancy between reviews was observed in terms of defining eHealth. For example, McLean et al [6] excluded Web-based tools and interventions for self-management in their Cochrane review on asthma patients because health care professionals were not actively involved with the ongoing delivery of the intervention. McGeary et al [22] chose a broader definition in their work on telehealth trials in pain management, including all studies that assessed a technology-based intervention extending care beyond the health care professional’s office.

Many authors did not conduct a meta-analysis because of important differences perceived in study populations, interventions and outcome measures [18,19,25,26,31-33, 35,36,39]. Instead, they performed a qualitative analysis of their findings. Several papers presented the results of a pooled analysis or subanalysis, despite substantial heterogeneity (ie, *I* ^*2*^ value >50) [6,12-14,23,24]. Three studies did not report heterogeneity [11,16,38].

### Trend Analysis

Since the publication by Ekeland et al in 2010 [9], the number of systematic reviews and meta-analyses on eHealth interventions in patients with somatic diseases has grown considerably (Table 7). In addition, 4 Cochrane reviews have recently been published [6,14,23,38]. However, a similar percentage of papers concluded that eHealth is effective/cost-effective or evidence is at least promising (65% vs 62%). Reviews focusing primarily on children or family caregivers still remain scarce. Between 2009 and 2012, home telemonitoring and video-teleconferencing were less frequently subject to a systematic review and/or meta-analysis on eHealth interventions, whereas educational tools and self-management programs were encountered more often. Data on economic outcome measures were less frequently reported in recent papers. Other study characteristics (eg, geographic area) barely differed between our review and the review by Ekeland et al [9].

**Table 7:** Trend analysis of differences in study characteristics of the current review compared with the review by Ekeland et al [9] published in 2010.

Study characteristic		Current review	Ekeland et al
Inclusion period, years		2009-2012	2005-2009
**Systematic reviews, n**		31	26
	Meta-analyses, n (%)	14 (45)	7 (27)
	Cochrane reviews, n (%)	4 (13)	0 (0)
**Study population, n (%)**			
	Children/adolescents only	3 (10)	0 (0)
	Family caregivers only	1 (3.2)	1 (3.8)
**Geographic area, n (%)**			
	Europe	15 (48)	17 (65)
	North America	18 (58)	18 (69)
	Asia	11 (35)	8 (31)
	Oceania	4 (13)	5 (19)
	Latin America	3 (10)	4 (15)
	Africa	0 (0)	0 (0)
	Not explicitly stated	13 (42)	8 (31)
**Authors’ conclusions, n (%)**			
	Effective/cost-effective	7 (23)	8 (31)
	Promising	13 (42)	8 (31)
	Limited/inconsistent	11 (35)	10 (38)
**Outcome measure, n (%)**			
	Health	28 (90)	24 (92)
	Costs	20 (65)	22 (85)
	Patient satisfaction	17 (55)	16 (62)
	Self-management	16 (52)	14 (54)
**Intervention components, n (%)**			
	Home telemonitoring	19 (61)	21 (81)
	Structured telephone support	10 (32)	12 (46)
	Video-teleconferencing	8 (26)	11 (42)
	Education	17 (55)	12 (46)
	Self-management programs	11 (35)	7 (27)
	Telerehabilitation	5 (16)	4 (15)
	Telemedicine (not otherwise specified)	1 (3.2)	0 (0)

## Discussion

The term eHealth can be defined briefly as the delivery of personalized health care at a distance through the use of technology. It is hypothesized that this field of medicine potentially enhances the quality of health care, with simultaneous reduction of health care costs. To support this hypothesis, we undertook a systematic review of systematic reviews and meta-analyses on the effectiveness/ cost-effectiveness of eHealth interventions in patients with somatic diseases. In addition, we performed a trend analysis to compare current findings with results from a systematic review by Ekeland et al published in 2010 [9].

In recent years, literature on eHealth has accumulated considerably. We found a total of 31 reviews, of which 20 (65%) concluded that eHealth interventions are effective/cost-effective or evidence is at least promising. Only 11 reviews (35%) showed no, limited, or inconsistent proof. These findings are consistent with the results from the review by Ekeland et al [9] (Table 7). Furthermore, trend analysis shows reviews focusing primarily on children or family caregivers still remain scarce. Although a pooled (subgroup) analysis of aggregate data from randomized studies was performed in a higher percentage of more recently published reviews (45% vs 27%), data on economic outcome measures were less frequently reported (65% vs 85%).

Because our review is a systematic review of systematic reviews and meta-analyses, it holds 2 important limitations. Firstly, we relied on the adequate inclusion and critical appraisal of individual studies, as well as on a correct interpretation of study results by the authors of the reviews and meta-analyses included in the current review. We did not investigate whether reviews on similar topics comprised identical studies; neither did we examine possible discrepancies in the analyses of these individual studies when included in more than one review or meta-analysis. Noteworthy, systematic reviews of systematic reviews have been conducted before in other fields of medicine, including reconstructive surgery and neuroradiology [43,44].

Secondly, reviews differed substantially in terms of study populations, intervention components, comparison groups, and outcome measures, for example. Therefore, it is difficult to identify which patients are likely to benefit from which specific intervention. Home telemonitoring and structured telephone support seemed to be effective/cost-effective in patients with CHF (Table 3), whereas evidence on both interventions seemed limited or inconsistent in patients with chronic pulmonary diseases (Table 5). Meta-analysis was often impeded because of heterogeneity among individual studies. This may have demanded careful conclusions from the authors of that particular review. In several reviews, a pooled (subgroup) analyses was presented despite substantial heterogeneity among individual studies (ie, *I* ^*2*^ value >50) [17,42]. Publication bias may have been the result of the exclusion of small individual studies with negative results, which could have ultimately lead to overestimation of benefits [45,46]. Noteworthy, Ciere et al [32] proposed methodological weaknesses may be partially because of artifacts of poor reporting, rather than being a reflection of poor study design or implementation.

Regarding the aforementioned methodological shortcomings, Ekeland et al [47] performed a systematic review in which they summarize methodologies used in research on eHealth interventions, discuss knowledge gaps, and postulate recommendations for methodological approaches for future research. Furthermore, we agree with recommendations made in previous reports to overcome the problem of between-study differences: researchers should adhere to and make transparent use of reporting guidelines appropriate for specific study designs. These guidelines may include Consolidated Standards of Reporting Trials (CONSORT)-EHEALTH for RCTs on eHealth interventions, Transparent Reporting of Evaluations with Nonrandomized Designs (TREND) and Strengthening the Reporting of Observational Studies in Epidemiology (STROBE) for observational studies in general, and Workgroup for Intervention Development and Evaluation Research (WIDER) recommendations for the reporting of behavioral interventions [32,48-51].

Because pilot schemes are often limited to fewer than 100 patients, many researchers in the past decade have advocated larger RCTs with standardized study designs to provide definite proof on the effectiveness/cost-effectiveness of eHealth interventions. Results of the recent Whole System Demonstrator trial—involving 3230 patients with diabetes mellitus, COPD, and CHF—showed that eHealth interventions are associated with lower mortality and emergency admission rates [52]. In our opinion, these results should provide an important stimulus to invest in the incorporation of eHealth in daily practice. However, implementation difficulties, such as resistant or refractory behaviors of health care professionals, are an international phenomenon [53]. The Normalization Process Theory (NPT), a sociological theory that provides a framework for understanding the relationship between technology and the social environment, has been used to develop implementation tools such as the eHealth Implementation Toolkit (E-HIT) [54,55].

Although large, well-designed RCTs are likely to further support the evidence on the effectiveness/cost-effectiveness of eHealth initiatives, we believe it is more desirable to focus on overcoming the problematic gap between pilot schemes and daily practice. As proposed in both reviews by Ekeland et al [9,47], formative process assessments and complexity studies can be further explored to achieve this goal.

In conclusion, the number of reviews and meta-analyses on the effectiveness/cost-effectiveness of eHealth interventions in somatic diseases has increased considerably in recent years. The majority of these papers show eHealth is effective/cost-effective, or at least suggests evidence is promising, which is consistent with previous findings. Data on economic outcome measures were less frequently reported in articles that were published more recently. This is an interesting finding, given the importance of formal cost analyses when considering implementation of eHealth interventions in daily practice. Although many researchers advocate larger, well-designed, controlled studies, we believe attention should be given to the development and evaluation of strategies to implement effective/cost-effective eHealth initiatives, rather than to further strengthen the evidence that has already been made available.

## Abbreviations

CHF: congestive heart failure
CONSORT: Consolidated Standards of Reporting Trials
COPD: chronic obstructive pulmonary disease
E-HIT: eHealth Implementation Toolkit
HbA1c: glycosylated hemoglobin
ICU: intensive care unit
LDL: low-density lipoprotein
NPT: Normalization Process Theory
PRISMA: Preferred Reporting Items for Systematic Reviews and Meta-Analyses
QUOROM: Quality of Reporting of Meta-analyses
RCT: randomized controlled trial
STROBE: Strengthening the Reporting of Observational Studies in Epidemiology
TREND: Transparent Reporting of Evaluations with Nonrandomized Designs
WIDER: Workgroup for Intervention Development and Evaluation Research
